# Data-Driven Visual Performance Analysis in Soccer: An Exploratory Prototype

**DOI:** 10.3389/fpsyg.2018.02416

**Published:** 2018-12-05

**Authors:** Alejandro Benito Santos, Roberto Theron, Antonio Losada, Jaime E. Sampaio, Carlos Lago-Peñas

**Affiliations:** ^1^Departamento de Informática y Automática, University of Salamanca, Salamanca, Spain; ^2^Department of Sports Sciences and Health, Universidade de Trás-os-Montes e Alto Douro, Vila Real, Portugal; ^3^Department of Sports Sciences, University of Vigo, Pontevedra, Spain

**Keywords:** football association, visual analytics, performance analysis, information visualization, collective tactical behavior

## Abstract

In soccer, understanding of collective tactical behavior has become an integral part in sports analysis at elite levels. Evolution of technology allows collection of increasingly larger and more specific data sets related to sport activities in cost-effective and accessible manner. All this information is minutely scrutinized by thousands of analysts around the globe in search of answers that can in the long-term help increase the performance of individuals or teams in their respective competitions. As the volume of data increases in size, so does the complexity of the problem and the need for suitable tools that leverage the cognitive load involved in the investigation. It is proven that visualization and computer-vision techniques, correctly applied to the context of a problem, help data analysts focus on the relevant information at each stage of the process, and generally lead to a better understanding of the facts that lie behind the data. In the current study, we presented a software prototype capable of assisting researchers and performance analysts in their duty of studying group collective behavior in soccer games and trainings. We used geospatial data acquired from a professional match to demonstrate its capabilities in two different case studies. Furthermore, we successfully proved the efficiency of the different visualization techniques implemented in the prototype and demonstrated how visual analysis can effectively improve some of the basic tasks employed by sports experts on their daily work, complementing more traditional approaches.

## Introduction

Soccer performance is a multifactorial process requiring high-level interaction analysis within physiological, technical, and tactical performances. At the elite level, technical staffs need to capture, process, and analyze great amounts of data, in order to measure performance in their respective teams and opponents, as well as assess potential prospects. Recently, the time pressure of this process and the constant increase in the amount of available data has demanded for a major emphasis in the visualization methods. In fact, technical staffs are nowadays expected to capture, process, analyze, and visualize data to provide fast assimilated information for coaching purposes. Current technology allows capturing data from players' positions, either in competition or training scenarios, with very acceptable degrees of accuracy. These technological advances can use radio frequency systems (Frencken et al., 2010), semi-automated computer vision systems (Di Salvo et al., 2006), or differential and non-differential GPS units (Varley et al., 2012). This GPS technology has contributed to change how performance analysis is carried out in outdoor and indoor team sports by providing objective and reliable data on player's activity. Technically, these systems use the earth-orbiting satellites that emit constant coded signals to track the position of a receiver (Larsson, 2003). Using the accurate positions from the receivers such systems can process real-time distance and speed. These measurement systems are portable and relatively less expensive when compared to any other systems. At the end of these processes, positional data containing the players' positions is obtained.

Research and practical applications resulting from positional data are much focused on the descriptions from the activity profiles, such as quantifying the physical and physiological demands of soccer players (Cummins et al., 2013; Abade et al., 2014). Although, these are certainly very important determinants of performance, recent research also focuses on describing collective tactical behavior aiming to reveal new insights and a better understanding of players' performances and soccer complexity (Gonçalves et al., 2014, 2018; Sampaio et al., 2014). There are several team and individual key variables suggested as key performance indicators to address the collective tactical behavior (Memmert et al., 2016). Nevertheless, their apparent utility is strongly diminished by the lack of any structured software tool capable of providing easy comprehension of such variables. In fact, data is usually processed using non-specific scientific-based software and therefore the task often becomes unbearable for soccer technical staffs. Sports analysis workflow requires tools and techniques that ideally would provide a way to effectively filter the data being analyzed on demand and display this data in a way that enhances the experts' analysis capabilities in the context of the problem. Under this scope, researchers could easily identify repeated patterns in the data over the course of a game and the global understanding of key events would be largely augmented.

## Problem Description

There is a great interest among Sports Science scholars in explaining how a group of players collectively perform at a given sports event. In the same way as it occurs in other areas of knowledge, researchers have turned their eyes to computers with the hope of obtaining valuable information that can help them to answer their particular research questions. As a consequence, computer-supported team tactics analysis is an emerging topic that has generated important publications in recent years. From a computational perspective, different authors have tried to explain collective behavior in sports employing diverse techniques such as statistical (Bialkowski et al., 2014), trajectory (Perin et al., 2013), or network analysis based on passes (Passos et al., 2011), among others—see (Gudmundsson and Horton, 2017) for a complete list. All these tools are specifically designed to capture and automate many of the tasks that are required to obtain satisfactory analyses and ensure a correct interaction between the analyst and the machine.

There are times however, when these techniques have not been implemented in an interactive application due perhaps to their novelty in the field. In these cases, the otherwise automated tasks must be manually performed by the analysts employing general-purpose scientific software such as *Matlab* or *Mathematica*, where the algorithms at play are run. The results of these algorithms are then captured in static charts, graphs, or pieces of text which can be crossed with other information that is available to the analyst such as in-game video recordings in order to form hypotheses. Using this new knowledge, the analyst may vary the input data (e.g., the players' positional data, analyzed period of time) and specific parameters of the algorithms and rerun the computations. He or she will repeat this procedure as many times as necessary in a series of progressive refinements until enough evidence to support or refuse a hypothesis has been found.

When these series of informational exchanges between the human and the computer have not been optimized, the whole process may become highly time-consuming, inefficient and frustrating for the user as some authors have pointed out in the past (Nielsen, 1993).

To the best of our knowledge, there are no examples of interactive tools in the literature that make use of movement patterns and complex inter-player coordination metrics to explain collective tactical behavior in soccer games (Sampaio and Maçãs, 2012). Therefore, our proposal tries to fill that void by offering the analyst an interactive dashboard that (a) exposes physical and inter-player metrics in a visual manner to the final user, (b) enables a spatiotemporal and multivariate analysis of the game and (c) generally reduces the cognitive load of the analysis task by ensuring an adequate interaction with the computer. Unlike other alternatives, our approach does not rely on the ball's position and therefore we did not consider it in our study, although we are aware of its usefulness in the analysis and therefore we plan to introduce it in further developments (we refer the reader to the final section for a full list of future lines of research).

## Key Metrics

The dynamic variables employed in the pilot tool and that serve as a basis for the expert analysis task are outlined in this section. Firstly, we present those regarding the collective aspect of the game, (i.e., they measure characteristics of a group of players). Later in this section the individual variables affecting single players are introduced. These variables are considered secondary and are meant to support the more complex group analysis of the game.

### Collective Analysis

#### Team Center

Also known as centroid, gravity center, or geometrical center. The centroid is the average position of the outfield players. It is calculated as the mean lateral and longitudinal position of all outfield players' coordinates at a specific time, each of them contributing equally to the computation (Sampaio and Maçãs, 2012; Folgado et al., 2014a; Gonçalves et al., 2014, 2016; Sampaio et al., 2014). Based on the mean point of players' locations, this variable captures oscillatory movements in a group of players such as movements toward or away from the goal lines or the sidelines (Duarte et al., 2012a,b). The centroid of the attacking team moves forward. The defending team tries to prevent the attacking team from scoring by moving backward. Thus, its centroid will move backward. It is employed to measure intra and inter team coordination in a group of players and it reveals important facts about team tactics and other valuable data for the analyst. The relative distance between opposing teams' centroid positions in the longitudinal direction (i.e., considering the distance to the goal) seems to be related to goal scoring opportunities (Frencken et al., 2011; Folgado et al., 2014b). From the centroid position of both teams three measures can be calculated: (i) distance to centroid, as measured by the absolute distance of each player to the geometric center of the team, plotted against time; (ii) distance to opponents' centroid, as measured by the absolute distance of each player from the geometric center of the opposite team, plotted against time; and (iii) distance between teams' centroids, as measured by the absolute distance between the geometric centers of the two teams plotted against time. Figure 2 presents a group of four players with its association centroid and radial distances to it.

#### Team Dispersion

Among the parameters related to the expansion and contraction of a group of players in a time interval is also worth noting the **Stretch Index**. It is defined as the average radial distance of the players' positions to the team centroid (Frencken et al., 2011; Folgado et al., 2014b). This variable captures the synergistic counter-phase relation of contraction and expansion behaviors of teams as a function of exchanges in ball possession. First derivate of this measure may also evidence the speed at which teams stretch or shorten their dispersion on the field (Duarte et al., 2012b). Several studies have validated the Stretch Index as a revealing parameter to interpret the dynamics of team tactics (Duarte et al., 2013; Travassos et al., 2014).

Another popular measure of Team Dispersion is the ***effective playing space or surface area***, calculated as the area of a polygon drawn by linking the externally positioned player in each team's formation (Gonçalves et al., 2017a). This variable expresses the relation between the shapes and the occupied spaces of the two teams, and how they change over time. Overlapped areas can also be obtained. The Surface Area of a group of players provides valuable information to the analyst regarding the portion of the field that is being occupied by the team while attacking or defending and it is intimately linked to the concept of stretch index previously presented. Derived from the later it can be measured the **team spread** (Ric et al., 2016), a value defined as the maximum difference between players' positions in the longitudinal—**team length**—and transverse—**team width**—axes. The team length captures the compactness of the whole team and its variation as a function of changes in performance constraints. It can be used in the monitoring of specific reference values for team length, or to evaluate depth differences between teams. When in defense, the width of a team may reveal the potential for the opponents to find inner or outer spaces to penetrate. When attacking, it may indicate the lateral spread of the team.

#### Team Synchrony

Recent studies employed phase analysis to identify inter-player coordination (Folgado et al., 2014a, 2015, 2018; Gonçalves et al., 2017a). This is a signal processing statistic that describes synchronization in a non-linear manner, by providing a quantitative measure of coordination between 2 oscillators. The relative phase calculation is done by using a Hilbert transform and the obtained values are expressed in angles. The values close to 0° correspond to simultaneous patterns of coordination, often referred to as moving in-phase, whereas relative phase values close to 180° correspond to asynchronous patterns of coordination, often referred to as moving in anti-phase (Sampaio and Maçãs, 2012). As tactical training is often directed to achieve symmetrical movements between individuals in certain attacking or defending situations, it is commonly used as an estimation of the degree of assimilation of such training by the athletes. Related to this approach, *ApEn* is also employed in this type of analysis. *ApEn* can be used to identify regularity in players' movement patterns (Sampaio and Maçãs, 2012; Silva et al., 2016; Gonçalves et al., 2017b). This is an adequate statistic to analyze non-linear time series data that holds both deterministic chaotic and stochastic processes. The *ApEn* algorithm quantifies regularity in a time series, by measuring the logarithmic likelihood that runs from patterns that are close (within tolerance wide r) to m contiguous observations and remain close (with the same r) on subsequent incremental comparisons. The obtained values are unitless real numbers ranging from 0 to 2, with lower values corresponding to more repeatable sequences of data points (Pincus, 1991; Stergiou et al., 2004; Harbourne and Stergiou, 2009; Yentes et al., 2013)

### Individual Analysis

It is frequent that players are responsible for specific pitch areas which they are supposed to control during the game, according to tactical schemes, game rules, and other constraints. Team behavior is an emergent result of many individual labors in interaction, each one performed in different parts of the game field and consequently, its analysis can be done in a geospatial manner. Heat maps suppose a simple yet useful way to visualize these phenomena, as several studies corroborate. As we will discuss in the next section, we use this technique to represent not only positional data for each player, but also changes in pace, which are of major importance in team sports.

## Proposed Workflow

**Figure 1:**
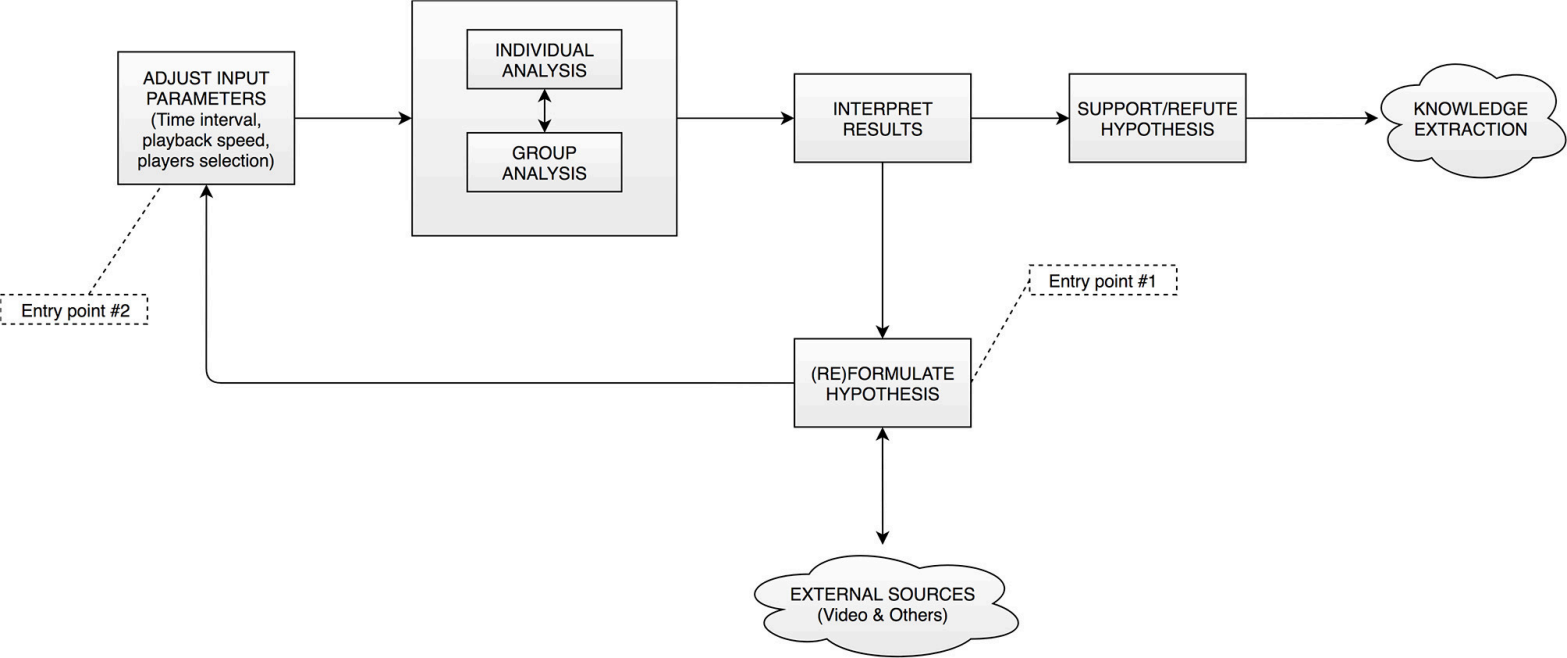
Proposed workflow. The user starts the analysis tasks at either of the two entry points to the tool.

According to the ideas introduced in section Problem Description, our visual analytics tool proposes an interactive workflow that seeks to accelerate current expert methodologies in the field and to assist the analyst in finding meaningful facts about group behavior contained in the positional data under study. There are two main operation modes for this workflow: In the first mode, the user starts with a known hypothesis that wants to validate (for example, the low level of synchronization between two defenders motivated a goal by the opposing team). In the second mode, the analyst explores the data without a previously known hypothesis and this hypothesis is formed once a particular event in the visualizations is spotted. (see Figure 1 and explanation below).

**Figure 2:**
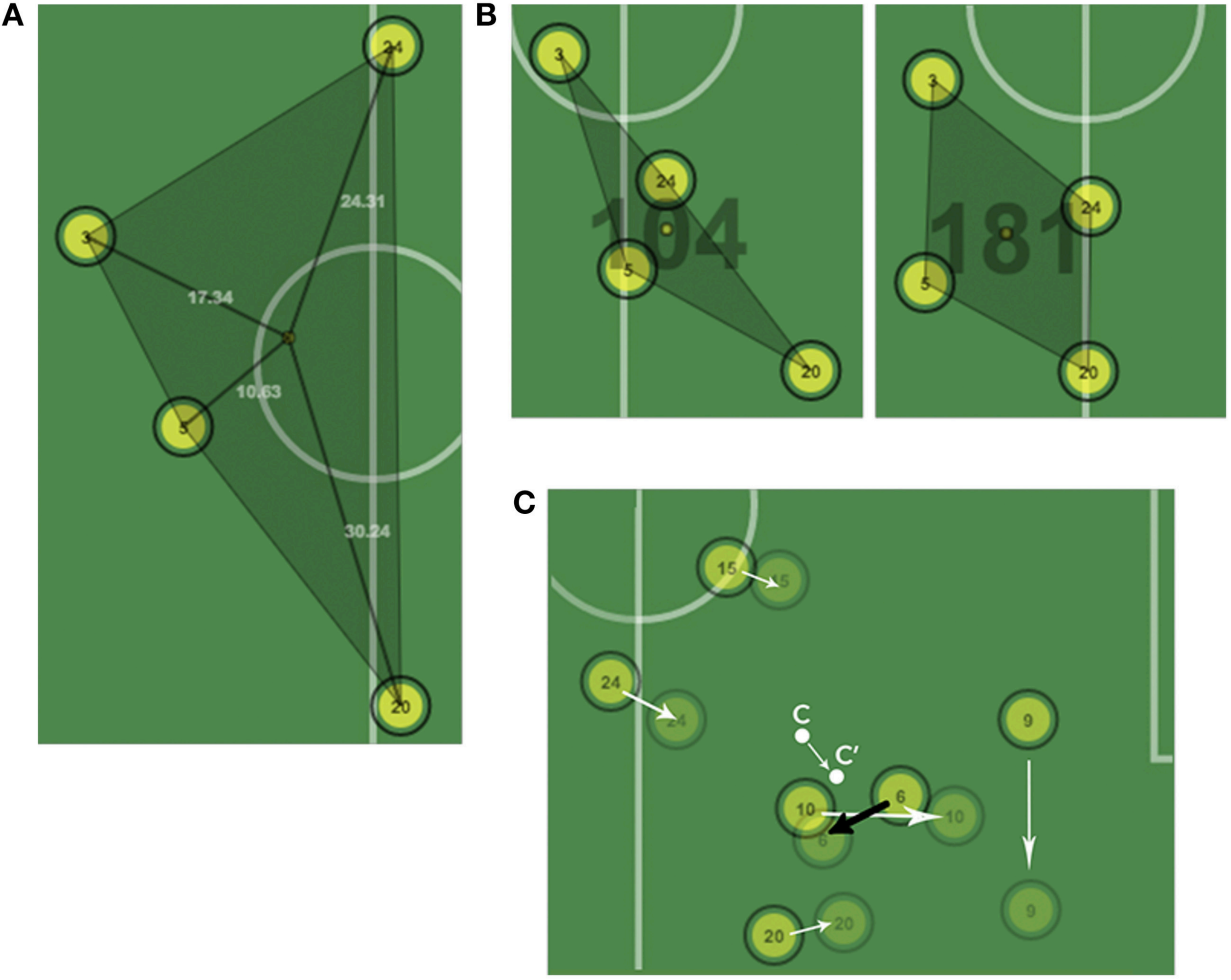
**(A)** Centroid of a group of players with radial distances. **(B)** The same group of players captured at two different instants of the game. Despite being equally dispersed, the players on the right covered a larger effective playing space. **(C)** Intra-group coordination in a series of movements. Player number 6 performs in the opposite direction of his group.

### Stage 1: Data Exploration

In this stage, the expert makes use of the application to identify key events in the game by measuring and comparing different multivariate attributes of an individual or a group of players. It starts by selecting the desired system configuration, which initially is based solely on expert knowledge and external resources (i.e., real in-game video and others). The selected interval can correspond to a whole period or to a specific section of interest (e.g., last 15 min). We have focused on analyzing the progression of attributes during a period of time and also on revealing significant variations of different variables to the analyst. Once evidence that supports a possible explanation of a key game event has been found, the analyst's attention is drawn to a specific slice of data (temporal interval, set of players, portion of the pitch). At this point of the workflow, a new set of key events and facts has been identified and the analyst can proceed to the second stage.

### Stage 2: Hypothesis Validation

At this stage, the analyst acquires knowledge to update his or her set of hypotheses, which will serve as input for the first stage of the new iteration. The analyst then tunes the system using new settings according to the hypothesis he or she wants to test, this including time intervals (usually a sub-interval of the previously selected time interval), virtual player playback speed, combination of visualizations, and a selection of players. More feedback from external sources can be incorporated to help in the selection of the configuration parameters in the new iteration. Once the system has been configured, the data is filtered accordingly to reflect the analysts' mental model in the investigation process.

The visualizations employed in this second approach usually correspond to a higher level of granularity and are oriented to allow effective detection of subtle variations in the observed parameters. Once this process has been completed, the analyst starts a new iteration by exploring data using the system, in a continuous cycle that will end with the final set of hypotheses being tested.

## Visual Analytics Interface

**Figure 3:**
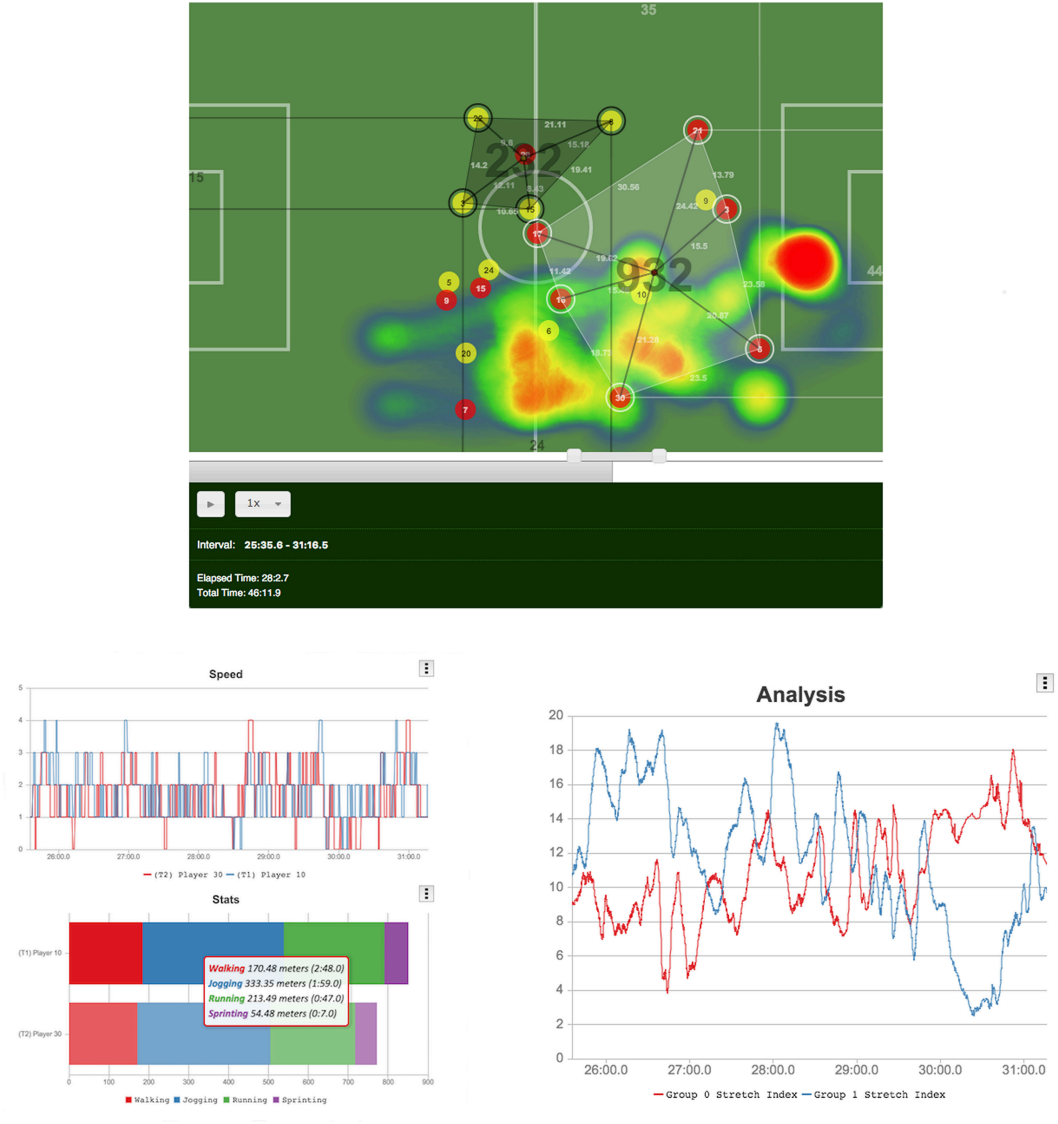
Top: Virtual player and group analysis. Bottom-**left**: A Comparison in the speed and distance covered by two different players. Bottom-**right**: Line chart showing the evolution of each group's stretch index in the selected period.

In Figure 3, we identify the three main components of our prototype: (1) The virtual player, that provides a real-time playback of the game and depicts the players' positions at all times. It serves the analyst to browse through the game looking for interesting in-game situations. As seen in the image, other representations of the different metrics are also embedded in the animation. (2) Position and speed analysis area, in which these values are presented in the form of two different charts; and (3) group analysis area, in which line charts corresponding to some of the metrics employed in the virtual player can be generated.

Our pilot tool gives analysts the chance of evaluating performance at both individual and group levels. Each level focuses on different characteristic parameters and metrics related to the nature of each type of analysis (group or individual). Individual analysis supports group analysis and is the key to provide efficient knowledge acquisition within the proposed tool and workflow, and also is a key step in the process of finding meaningful game plays or explanations for observed group behavior. One of the major achievements of the pilot tool is to be successful in visually exposing the links between group and individual traits that otherwise would remain hidden to the analyst's eyes.

According to the collective characteristics previously explained in this paper, great emphasis was placed on producing visualizations that could reliably represent such traits by means of a combination of (1) animations; (2) graphs, and (3) glyphs. We considered of great importance to help the user to connect specific elements in these visualizations to real game situations and vice versa. In a similar way, it is also relevant to establish bidirectional relationships between group and individual traits, in an attempt to explain phenomena of interest occurring in the teams' dynamics and tactics. The visuals in the application follow were designed to effectively represent the concept of shared cognition in a group and how it affects to the individuals conforming it. In the following paragraphs, we discuss the different representations given for each group metric explained in section Key Metrics, and how the proposed visualizations effectively enhance the analyst's knowledge acquisition process.

### Team Center

The process of selecting the players can be done using expert knowledge based or other individual metrics obtained in exploratory analysis. Based on these assumptions our example application allows the analyst to freely compose the study groups in the way he or she prefers, being the only constraint the minimum number of players that can conform a group. This way we enable the expert to focus only on the relevant individuals involved in a game play (i.e., 3 forwards vs. 4 defenses) and thus we effectively remove unwanted positions that could alter the resulting team center. In the case studies proposed at the end of this paper we elaborate further on this aspect.

### Stretch Index

In the proposed animation, players' distances are drawn as a line linking the centroid with the correspondent positions along with its numerical value (in meters) in a similar fashion to the surface area representation. By interacting with the interval selection handle, the analyst can select a game play of his or her interest or a whole period. The stretch index for the two groups of study is calculated and immediately presented on the analysis pane of the tool for further comparison. As we will see in the upcoming sections, these measures are especially important for group coordination studies. In our implementation, team center is intimately linked to the surface area covered by the group, and, in particular to the convex hull resulting of the players' positions, that is presented in the next section.

### Surface Area

Given that this animation is updated every frame according to the changes in the different positions we chose the Graham Scan algorithm to perform the convex hull computation because of the following two reasons: (a) it performs in logarithmic time *O(n log n)* and (b) the algorithm is especially well-suited for calculations that involve a small amount of points, as is our case (maximum 10 points for 10 different players). In our proposed animation, we draw a shadow polygon to remark the area that is being occupied by the team at a certain time. We also project the points to the real field measures, so we can also present the value in square meters (m2) drawn on top of the polygon's centroid. Furthermore, we represent inter-player distances in a similar way to what we did for radial distances in the previous section. These representations can be shown or hidden up to the analyst's discretion, following the approach taken in the rest of the example application.

### Team Length and Width

In our implementation, we use the four sides of the field as planes in which the two polygons corresponding to the two groups will be projected. First group will employ the left and bottom sides and the second group will make use of the upper and right ones. We also draw numerical values, so the analyst is informed of the real measure at all times.

### Team Synchrony

In previous sections we measured team coordination by applying phase analysis to two or more oscillating functions. Once an analysis group has been established and the time interval selected, the pilot tool is able to compute time-dependent functions from the continuous values given by the distances to the centroid of the group of each player, which can be added to the phase calculation. As a consequence, the analyst is able to explore how coordination between pairs evolves in such given period. The tool also offers the possibility to calculate the *ApEn*, a measure that informs on the unpredictability of the phase. High values represent a more chaotic coordination in the movements of the players in relation to the centroid.

### Individual Analysis

It is of major interest for analysts to inspect traits related to certain players in the group when searching for events that could justify certain group behaviors. In our work flow, the expert can compare information from these two different sources in a visual manner to speed up the finding of meaningful conclusions as opposed to traditional, manual techniques.

### Speed Evolution Chart

Given the different players' positions and their evolution in time we are able to calculate the speed, which is later discretized in five intervals or paces, represented in Table 1. Discretization of the speed variable occurs only once per match data set and in the data import phase. During an analysis session, the user can add players to the individual analysis zone using the mouse secondary button.

**Table 1:** Description from the speed intervals used in the application.

**Pace**	**Speed Interval**
Standing	Less than 0.2 m/s
Walking	Between 0.2 and 2.1 m/s.
Jogging	Between 2.1 and 3.8 m/s.
Running	Between 3.8 and 6.1 m/s.
Sprinting	More than 6.1 m/s.

### Change of Pace Chart

This line chart, reflects the different changes of pace for one or more players in the given analysis interval. The analyst can browse through the data evaluating the speed progression in the selected time interval. In line with other visualizations presented in this paper, this chart is also interactive in the way that it allows the researcher to navigate to the detected key event in the virtual player, effectively reducing the cognitive load that is involved in the analysis process.

### Distance Covered Comparison Chart

We employ a stacked bars chart to allow fast visual comparison of the distance covered by players in a time interval. Each stack represents the portion of the total distance performed at each one of the different speed intervals presented above. This enables the analyst to have a bird's eye perspective of the effort performed by the different players in a group, giving the analyst the perception about the time periods in which a player performed at a certain pace.

### Heatmaps

Spatial distribution maps or heat maps are a popular means to visualize spatio-temporal variables such as position or speed. As a companion visualization for the stacked bars chart, the analyst also has the possibility to generate this kind of visualization on demand. By employing heatmaps we are able to provide a better picture of a player's performance through any given period of time.

### Distance to a Point

Finally, the pilot tool allows analysts to make in-map measures among one or more players to a specific coordinate point in the field, as shown in Figure 3.

## Case Studies

A real elite soccer match was examined to demonstrate the capabilities of the proposed pilot tool. Positional data of the two teams was acquired using player visual tracking at 10 Hz resolution, resulting in ~27,000 coordinate entries per player per time. Firstly, a significative event—in this case a goal-, which is known by the user prior the analysis task, is presented. Then the prototype is used to search for other secondary events within the relevant play build-up that support a certain hypothesis that explains the final outcome of the play. The other example proposes a free exploration of the dataset. By employing phase analysis, the analyst is able to identify significative events that generate different kinds of situations of interest in the game.

### Case Study #1: Analyzing a Goal Scored Build-up Play

**Figure 4:**
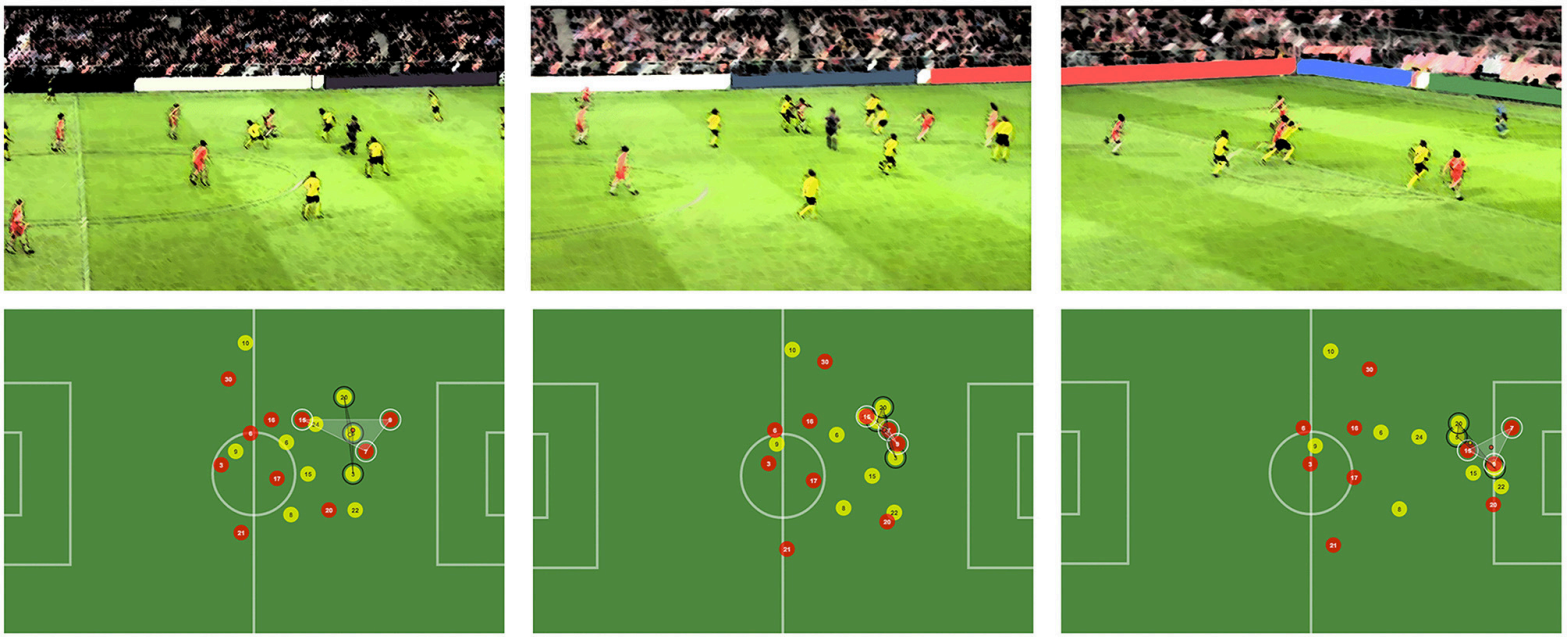
Build-up phases of a scored goal. The area covered by attackers and defenders is depicted in the system.

As an operational example, Figure 4 includes the original video footage and the two-dimensional visual representation as seen in the tool of three different moments of a scoring play. The key players intervening in the play are three attackers of the yellow team and three defenders of the red team. Covered area by the two groups is shown in the tool. At the center of each area there is a dot filled with the color of the team. This allows us to know where the centroid of the three players is, allowing the user to see their average positions at all times. It is known there is an increase in the chances of scoring a goal when the attacking group's centroid surpasses that of the defenders. In this use case, we verify how this technical event can be easily detected in our pilot tool to explain a real game event.

The first moment depicted in Figure 4 shows the start of the build-up, where Player 15 (red) gets the ball in the offense phase. In the second moment, he has surpassed a midfielder and is approached by two of the three highlighted defenders, which creates an opportunity for Player 7 (red) to get a scoring chance. In the last moment, Player 15 has already passed the ball to Player 7, who will finish easily and score a goal due to the defenders being stuck in more advanced positions, making it impossible to catch him. The second moment represents the key to the scoring chance and ultimately the goal. By exploring the positioning data, we better understand the impact of the two defenders (20 and 5, yellow) on the play. They decide to close onto the attacker with the ball (15, red) and that causes the centroids of both groups to get at practically the same position. What is more, this triggers an unbalanced situation in which players 9 and 7 of the attacking team (red) are left alone against Player 3 of the defending one (yellow). When the two defenders that tried to prevent the pass, start recovering and running back, it is late enough for Player 7 (red) to be on a clear scoring position, as depicted in the third moment. The difference between both centroids at this point is the largest of the sequence. This fact is in line with the proposals from other authors previously explained (Duarte et al., 2013), since from the moment the average position of the attackers was more advanced than that of the defenders, the play went their way and they ended up scoring.

**Figure 5:**
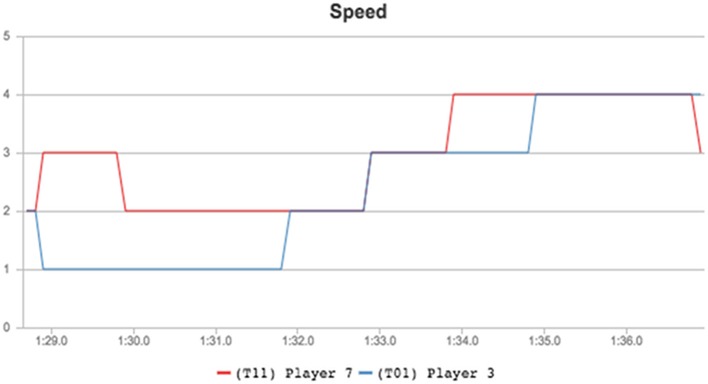
Speed comparison between players 7 (attacking) and 3 (defending) during the analyzed play.

Further explanatory facts can be found using individual analysis, for example if the speed evolution graphs of two of the players are considered: Player 7 (attacker, red) and Player 3 (defender, yellow). As depicted in Figure 2, Player 7 maintained a higher speed over Player 3 during the 8 analyzed seconds. The most important moments stand between the 1:34 and 1:35 marks, as Player 7 sprinted toward the goal a second after the defender started his movement. This allowed him to gain sufficient advantage to be alone in a scoring position later, as seen in the last moment depicted in Figure 5.

### Case Study #2: Studying Inter-player Coordination With Relative Phase Analysis

**Figure 6:**
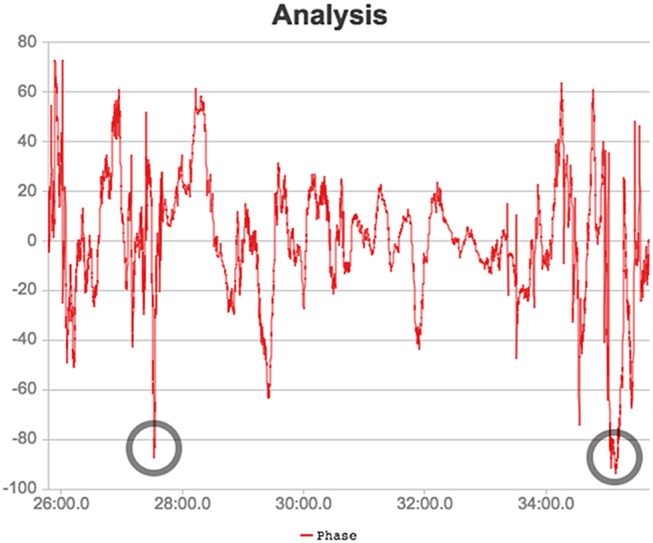
Phase analysis for the two defenders between minutes 26 and 38 of the second half. The two lowest moments of coordination are rounded in the image.

Relative phase can also be used to identify inter-player coordination. Two instants between minutes 28 and 38 are highlighted in Figure 6 depicting the times when the coordination between Player 3 and 6 of the red team (defending) was low. This implies that one defender moved toward the centroid while the other moved away from it, creating an unbalanced situation in the defense which has important implications on the outcome of the play (for example the first instant corresponds to a goal score by the opposing team).

**Figure 7:**
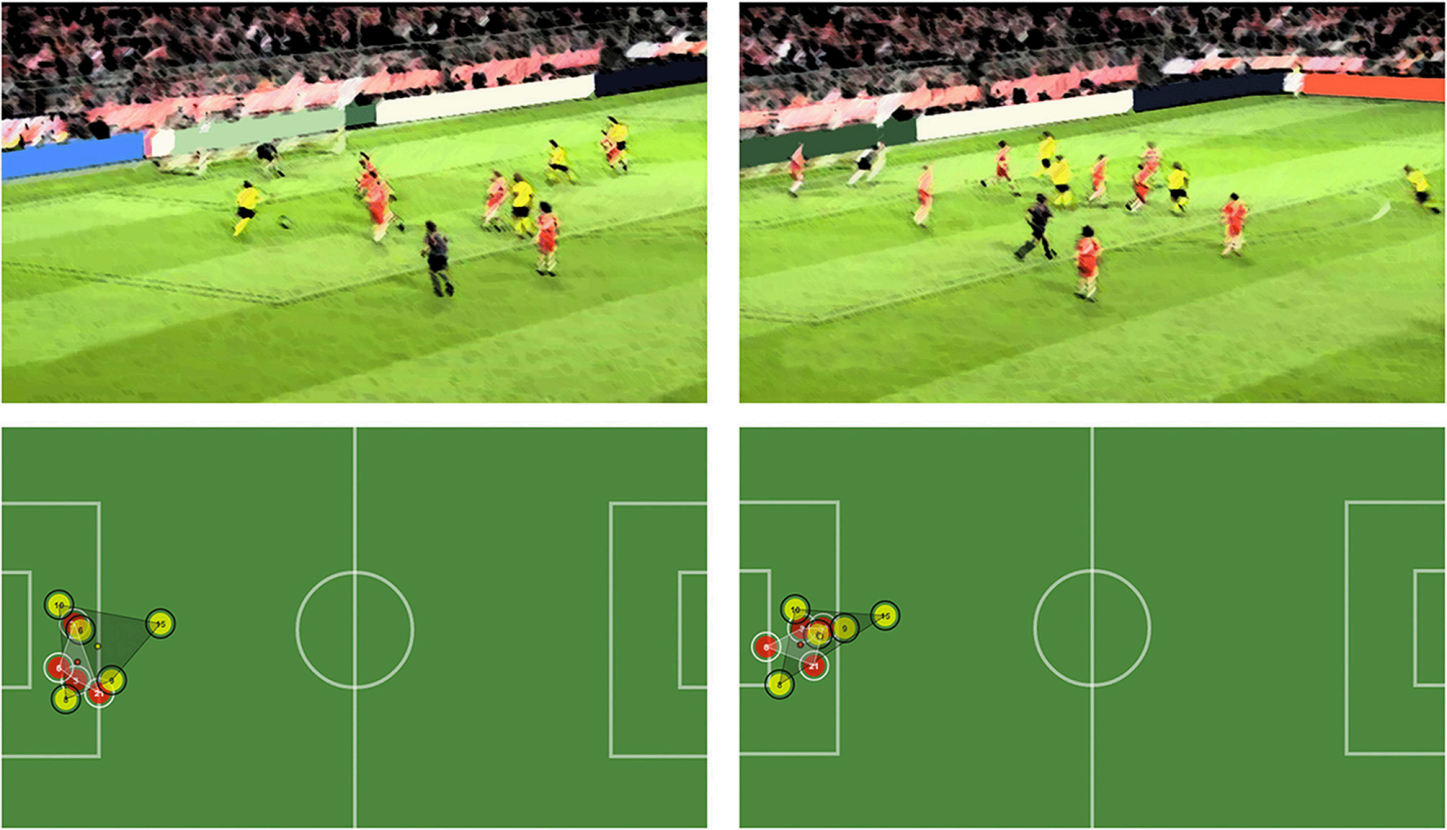
First moment of low coordination between two defenders (Players 3 and 6, red). The outcome is a goal by the attacking team.

The two instants of the sequence are depicted in Figure 7. By reproducing the play, we can see how the two defenders go back to cover Player 8 (yellow) as he surpasses them, trying to prevent a scoring chance. This attacking player then passes the ball back and that causes the defenders to lose their coordination, as one stays still at the goal line and the other runs to prevent the shot from the attacking player (number 15, yellow) who is outside of the goalkeeper's area. Moments of low coordination between defenders of the red team are generated in situations where the defenders are close together and an unexpected action (such as a back pass) happens. This unpredictable situation forces the defenders to lose synchronization with the rest of his teammates meaning each one of them took different directions, as it happened in the presented situation: In this concrete case, one decided to stay put in the goal line and the other to cover the shot, thus creating completely opposite movements from the centroid of the whole defense.

**Figure 8:**
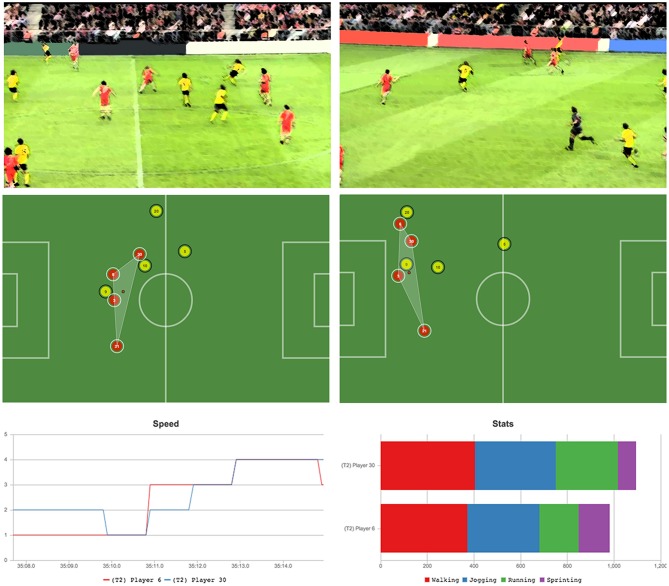
Top: Second moment of low coordination between two defenders (players 3 and 6, red). Bad defending from another player causes the disruption. Bottom: Speed and movement comparison between the two defenders involved in the opposition advantage play.

The second point of low coordination is depicted in Figure 8. In this case, the play did not result in a scoring chance, although we comment on other interesting aspects of the game that generated this situation. In this play, player 5 passes the ball to Player 20 (both attacking, in yellow), and Player 30 (defending, red) is not fast enough to stop the attack, making Player 6 (red) to leave its defense position to cover the area left by his team mate. It becomes clear that bad defense by Player 30 caused a break in the coordination of both central defenders (3 and 6, red). While Player 3 had to remain covering the attacker (Player 9, yellow), Player 6 had to go out of his zone of influence to stop Player 20 and prevent a cross or any other potentially dangerous play on offense. This special situation could also be easily identified in the pilot tool by employing the line graph, without the need to visualize the entire 6 min of game.

## Conclusions

In this study, we demonstrate that including visual analysis techniques in sports group behavior analysis can produce both high quality results and a satisfactory experience with the computer at the same time (see Supplementary Video 1). We designed an iterative workflow companion to this tool that effectively captures and accelerates many expert workflows that had to be performed manually or semi-automatically in the past. We found that the application of data visualization and user-centered design techniques helps to maintain the overall learning curve of the system flat without compromising the accuracy or efficiency of the analysis. In the provided examples, we could also appreciate how the proposed workflow can scale to many different game situations and combinations of analysis types -collective or individual. By following a top-down approach that goes from the general to the specific, the analyst can navigate the positional data by progressively tuning the interface controls and reach to conclusions of interest. Furthermore, the advantages of combining the proposed workflow with a visual tool could be noticed in the implementation of two different use cases that employed geospatial data acquired from a professional match in a very similar setting to what many sports scientists have in their daily work.

Despite of these promising first results, the ideas presented in this paper require of further validation and more complex user studies that we expect to implement in future research, along with the following lines of future work that we compiled during our research:

### Positional Data of the Ball

As it has been commented before in this paper, some authors take the ball position into account when calculating different group metrics. Given the recent advances in positioning technologies, we expect to be able to work with more data sets that include this feature.

### Social Network Analysis

As a result of the incorporation of the ball position, we plan to generate social network visualizations that depict associations of players during the game, extracting statistical data related to the number of passes between players, direction of the pass and other traits, which will be used as inputs for our system. This information should be presented to the user by employing dynamic network analysis and other complex network visualization techniques.

### Visualization of Biomechanical Variables

In our current implementation, we discretize the speed variable according to five predefined clusters. In a similar way, we could employ other non-geospatial variables gathered using different equipment attached to the athlete's body, such as a pulsometer.

### Visualization of Labor Division and Areas of Influence

Different studies divide the effective playing space of a group of players in several areas of influence (one per player). There are previous attempts to achieve this functionality in visual terms by making use of Delaunay triangulations and Voronoi cells.

### Real Video Playback

The pilot tool is able to link incoming metrics data with real in-game situations by establishing an interactive relationship between the virtual player and the two analysis zones. During analysis sessions, analysts tend to complement the usage of these prototypes with real video playback of the game under study. Currently this process must be performed manually, but it would be interesting to reduce the number of steps needed to reach to relevant parts of the video in a similar way to how the virtual player performs in the current tool.

## Author Contributions

RT, JS, and CL-P: Conceptualization; JS and CL-P: Funding acquisition; AB, RT, JS, and CL-P: Methodology; CL-P: Project administration; AB, RT, and AL: Software; AB, RT, and AL: Visualization; AB, RT, AL, JS, and CL-P: Writing—original draft.

### Conflict of Interest Statement

The authors declare that the research was conducted in the absence of any commercial or financial relationships that could be construed as a potential conflict of interest.

## Supplementary Material

Supplementary Video 1 Overview video. In this video, we present the proposed prototype to the interested reader. It gives an overview of all the features presented in this paper and depicts the usage of the tool to perform individual and group visual analysis employing real in-game data.
